# Patients with Exon 19 Deletion Were Associated with Longer Progression-Free Survival Compared to Those with L858R Mutation after First-Line EGFR-TKIs for Advanced Non-Small Cell Lung Cancer: A Meta-Analysis

**DOI:** 10.1371/journal.pone.0107161

**Published:** 2014-09-15

**Authors:** Yaxiong Zhang, Jin Sheng, Shiyang Kang, Wenfeng Fang, Yue Yan, Zhihuang Hu, Shaodong Hong, Xuan Wu, Tao Qin, Wenhua Liang, Li Zhang

**Affiliations:** Sun Yat-sen University Cancer Center, State Key Laboratory of Oncology in South China, Collaborative Innovation Center for Cancer Medicine, Guangzhou, China; Seoul National University, Republic of Korea

## Abstract

**Backgrounds:**

It has been extensively proved that the efficacy of epidermal growth factor receptor-tyrosine kinase inhibitors (EGFR-TKIs) is superior to that of cytotoxic chemotherapy in advanced non-small cell lung cancer (NSCLC) patients harboring sensitive EGFR mutations. However, the question of whether the efficacy of EGFR-TKIs differs between exon 19 deletion and exon 21 L858R mutation has not been yet statistically answered.

**Methods:**

Subgroup data on hazard ratio (HR) for progression-free survival (PFS) of correlative studies were extracted and synthesized based on random-effect model. Comparison of outcomes between specific mutations was estimated through indirect and direct methods, respectively.

**Results:**

A total of 13 studies of advanced NSCLC patients with either 19 or 21 exon alteration receiving first-line EGFR-TKIs were included. Based on the data from six clinical trials for indirect meta-analysis, the pooled HRTKI/chemotherapy for PFS were 0.28 (95% CI 0.20–0.38, P<0.001) in patients with 19 exon deletion and 0.47 (95% CI 0.35–0.64, P<0.001) in those with exon 21 L858R mutation. Indirect comparison revealed that the patients with exon 19 deletion had longer PFS than those with exon 21 L858R mutation (HR19 exon deletion/exon 21 L858R mutation  = 0.59, 95% CI 0.38–0.92; P = 0.019). Additionally, direct meta-analysis showed similar result (HR19 exon deletion/exon 21 L858R mutation  = 0.75, 95% CI 0.65 to 0.85; P<0.001) by incorporating another seven studies.

**Conclusions:**

For advanced NSCLC patients, exon 19 deletion might be associated with longer PFS compared to L858 mutation at exon 21 after first-line EGFR-TKIs.

## Introduction

Lung cancer, mainly non-small-cell lung cancer (NSCLC), remains to be the leading cause of cancer-related mortality worldwide [1]. Unfortunately, few treatment options are available for the majority of patients with advanced or metastatic disease [2]. In spite of marginal improvement in survival, most advanced patients require systemic therapy [3]. Recent advances in genetic discoveries have proved that EGFR-dependent pathway is activated in more than half of the patients with NSCLC and it plays an important role in the development and the progression of epithelial cells [4]. Small-molecule tyrosine kinase inhibitors (TKIs), including gefitinib and erlotinib, which specifically block the EGFR-dependent pathway, were the first targeted drugs to enter the clinical use for the treatment of lung cancer [5]. It has been extensively proved that NSCLC patients harboring sensitive EGFR mutations, which mainly refer to exon 19 deletions or L858R substitution in exon 21, usually benefit more from EGFR-TKIs than wild-type patients [6], [7]. However, whether the efficacy of EGFR-TKIs varies among different sensitive EGFR mutations is still controversial. Several studies have reported that advanced NSCLC patients with EGFR exon 19 deletion had a longer overall survival (OS) and/or progression-free survival (PFS) following treatment with gefitinib or erlotinib compared with those with the L858R mutation [8], [9], [10], but this result has not been shown in all reports [12], [13], [14], [15], [16].

Based on these scattered data, it is not convincing to conclude that a specific EGFR mutation may affects the response to EGFR-TKIs. Therefore, we sought to perform a meta-analysis by incorporating relevant studies to evaluate whether the clinical outcome differs between exon 19 deletion and exon 21 L858R mutation in advanced NSCLC patients treated with first-line EGFR-TKIs.

## Methods

### Literature search

All relevant articles were retrieved by searching through PubMed, Embase and the Central Registry of Controlled Trials of the Cochrane Library, using a combination of the terms “EGFR”, “epidermal growth factor receptor”, “tyrosine kinase inhibitors”, “TKI”, “exon”, “mutation”, “non-small-cell lung cancer” and “NSCLC”. An additional search through Google Scholar and a manual search through reference lists of pertinent reviews and included studies were performed. Two authors (FW and KS) carried out the search independently. No language or date restrictions were set in the search.

### Inclusion and exclusion criteria

Eligible studies should meet the following criteria: (i)clinical trials or retrospective studies which investigated the local advanced or metastatic (IIIB or IV) stage NSCLC with first-line monotherapy of EGFR-TKIs (e.g. gefitinib, erlotinib or afatinib) (ii) clinical trials or retrospective studies with a subset of NSCLC patients with specific sensitive EGFR mutation (exon 19 deletion or exon 21 L858R mutation); (iii) EGFR mutation analysis was performed on available tumor tissue samples instead of circulating free DNA in serum; (iv) prior neoadjuvant or adjuvant chemotherapy in patients with recurrence after surgery was permitted if it had elapsed from last administration to relapse at least 6-month; (v) hazard ratios (HRs) of EGFR-TKIs compared to conventional chemotherapy for progression-free survival (PFS) and HRs of exon 19 deletion compared to exon 21 L858R mutation for PFS in terms of EGFR-TKIs were available. Studies failing to meet the inclusion criteria will be excluded.

### Outcomes measures, data extraction and quality assessment

The clinical outcome for this meta-analysis was PFS. The data collection and assessment of methodological quality followed the QUORUM and the Cochrane Collaboration guidelines (http://www.cochrane.de). Data of PFS were extracted as HR and its 95% confidence interval (CI) from subgroup analysis by two investigators (YY and FW) independently. We used median PFS and the *P*-value to calculate the HR and its 95% CI then reviewed them with Review Manager (version 5.1 for Windows; the Cochrane Collaboration, Oxford, UK) if they were not displayed directly. The following main information was also extracted from included studies: author, year, trial name, details of therapeutic regimens, and number of participants with either 19 or 21 exon alterations. Three reviewers (YY, KS and FW) used the Jadad scale to assess the quality of included random control trials (RCTs) and a modified Newcastle-Ottawa scale to assess other studies. Discrepancies were discussed by all investigators to reach a consensus.

### Statistical analysis

Pooled HRs for PFS with 95% CI were calculated. Heterogeneity across studies was assessed with a forest plot and the inconsistency statistic (I^2^). The random-effects model was employed in case of potential heterogeneity and to avoid underestimation of standard errors of pooled estimates in direct meta-analyses as well as subsequent indirect comparison. All calculations were performed using STATA 11.0 (StataA Corp, College Station, TX). Based on the assumption that no significant difference of chemotherapy efficacy existed between exon 19 deletions and L858R mutations, we calculated the adjusted indirect comparison using the following formulas as previously described [17]. The log hazard ratio (log HR) of the adjusted indirect comparison for arm A versus arm B was estimated by 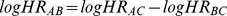, and its standard error for the log HR was 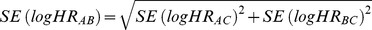, in which log HR_AC_ was the log HR for the direct comparison of patients with exon 19 deletion who received EGFR-TKIs versus those who received chemotherapy and log HR_BC_ were the log HR for the direct comparison of patients with exon 21 mutation who received EGFR-TKIs versus those who received chemotherapy. SE (log HR) was the standard error of the log HR for the direct comparisons. A HR value of less than 1 standed for patients receiving EGFR-TKIs with exon 19 deletion demonstrated longer PFS than those with exon 21 mutation. All CIs had two-sided probability coverage of 95%. A statistical test with p value less than 0.05 was considered as significant.

### Publication bias

An extensive search strategy was made to minimize the potential of publication bias. Graphical funnel plots were generated to visually assess a publication bias. The statistical methods used to detect funnel plot asymmetry were the rank correlation test of Begg and Mazumdar and the regression asymmetry test of Egger [18], [19].

## Results

### Eligible studies

**Figure 1:**
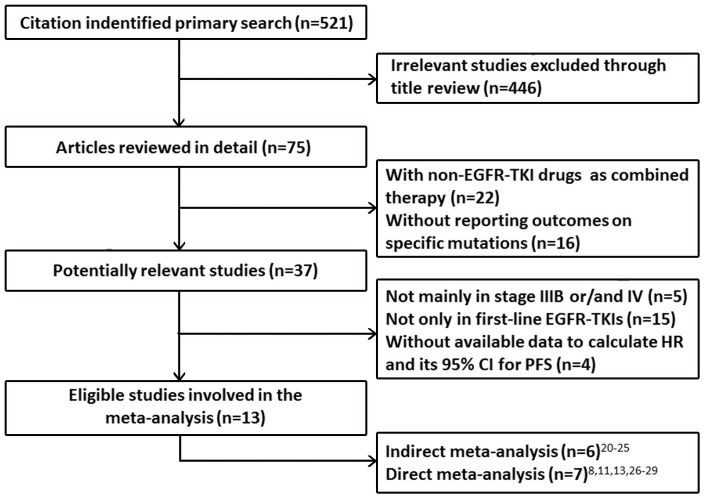
Profile summarizing the trial flow. CI  =  confidence interval; EGFR  =  epidermal growth factor receptor; HR  =  Hazard ratio; PFS  =  progression-free survival; TKI  =  tyrosine kinase inhibitor.

We identified 521 records according to the search strategy and focused on 13 studies which reported advanced NSCLC patients with either 19 or 21 exon alteration who received first-line monotherapy of EGFR-TKIs. Six phase III RCTs (IPASS [20], WJTOG3405 [21], OPTIMAL [22], EUTRAC [23], LUXLUNG3 [24], and LUXLUNG6 [25]), involving 1382 advanced NSCLC chemo-naïve patients, investigated the efficacy of EGFR-TKIs (gefitinib, erlotinib or afatinib) and conventional platinum-based chemotherapy. PFS and subgroup analyses of different sensitive EGFR mutations were reported. Therefore, these studies were selected for indirect meta-analysis. Another seven studies (clinical trials or retrospective studies) [8], [11], [13], [26], [27], [28], [29] involving 549 advanced NSCLC patients receiving first-line EGFR-TKIs (gefitinib or erlotinib) presented direct comparison of exon 19 deletion and L858R mutation for PFS. We incorporated these 7 studies for direct meta-analysis. **Figure 1** summarizes the flow chart. **Table 1** and **Table 2** summarize the characteristics of involved studies for indirect meta-analysis and direct meta-analysis, respectively.

**Table 1:** Characteristics of included studies for indirect meta-analysis.

Lead author (y)	Trial name (phase)	Therapeutic regimen of TKI	Therapeutic regimen of Chemo	Exon of EGFR mutation^a^	Sample size (TKI/Chemo)	HR_TKI/chemotherapy_ for PFS (95% CI)
Mok TS (2009)	IPASS (III)	Gefitinib 250 mg/d, po	Paclitaxel 200 mg/m^2^, d1, iv, q3w + carboplatin	19	140 (66/74)	0.38 (0.26–0.56)
			(AUC = 5–6) d1, iv, q3w×6 cycles	21	111 (64/47)	0.55 (0.35–0.87)
Mitsudomi T (2010)	WJTOG3405 (III)	Gefitinib 250 mg/d, po	Docetaxel 60 mg/m^2^, d1, iv, q3w + cisplatin 80	19	87 (50/37)	0.453 (0.268–0.768)
			mg/m^2^, d1, iv, q3w×3–6 cycles	21	85 (36/49)	0.514 (0.294–0.899)
Zhou CC (2011)	OPTIMAL (III)	Erlotinib 150 mg/d, po	Gemcitabine 1000 mg/m^2^, d1,8, iv, q3w +	19	82 (43/39)	0.13 (0.07–0.25)
			carboplatin (AUC = 5) d1, iv, q3w×4 cycles	21	72 (39/33)	0.26 (0.14–0.49)
Rosell R (2012)	EUTRAC (III)	Erlotinib 150 mg/d, po	Docetaxel 75 mg/m^2^, d1 or gemcitabine	19	115 (57/58)	0.30 (0.18–0.50)
			1000–1250 mg/m^2^, d1,8, iv, q3w + cisplatin 75			
			mg/m^2^, d1 or carboplatin (AUC = 5–6) d1, iv,	21	58 (29/29)	0.55 (0.29–1.02)
			q3w×4 cycles			
Sequist LV (2013)	LUXLUNG3 (III)	Afatinib 40 mg/d, po	Pemetrexed 500 mg/m^2^, d1, iv, q3w + cisplatin	19	170 (113/57)	0.28 (0.18–0.44)
			75 mg/m^2^, d1, iv, q3w×≤6 cycles	21	138 (91/47)	0.73 (0.46–1.17)
Wu YL (2014)	LUXLUNG6 (III)	Afatinib 40 mg/d, po	Gemcitabine 1000 mg/m^2^, d1,8, iv, q3w +	19	186 (124/62)	0.20 (0.13–0.33)
			cisplatin 75 mg/m^2^, d1, iv, q3w×≤6 cycles	21	138 (92/46)	0.32 (0.19–0.52)

a Exon of EGFR mutation means either exon 19 deletion or exon 21 L858R mutation.

Abbreviations: AUC  =  area under the concentration time curve; Chemo  =  chemotherapy; CI  =  confidence interval; EGFR  =  epidermal growth factor receptor; HR  =  Hazard ratio; PFS  =  progression-free survival; TKI  =  tyrosine kinase inhibitor.

**Table 2:** Characteristics of included studies for direct meta-analysis.

Lead author (y)	Study (phase)	Therapeutic regimen of TKI	Exon of EGFR mutation^a^	Sample size	Median PFS (m)	*P*-valueof PFS	HR_19/21_ of TKI^b^ for PFS (95% CI)
Maemondo M (2010)	NEJ002 (III)	Gefitinib 250 mg/d, po	19	58	11.5	0.90	0.939 (0.3518–2.5061)
			21	49	10.8		
Asahina H^c^ (2006)	Prospective (II)	Gefitinib 250 mg/day, po	19	13	8.3	0.678	1.410 (0.2785–7.1388)
			21	3	11.7		
Jackman DM^c^ (2006)	Retrospective	Gefitinib 250 mg/day, po or erlotinib 150 mg/day, po	19	22	24	0.04	0.417 (0.1810–0.9608)
			21	10	10		
Li JJ (2012)	Retrospective	Gefitinib 250 mg/day, po or erlotinib 150 mg/day, po	19	33	9.0	0.002	0.778 (0.6635–0.9123)
			21	21	7.0		
Lee VHF (2013)	Retrospective	Gefitinib 250 mg/day, po or erlotinib 150 mg/day, po	19	64	12.8	0.040	0.649 (0.416–0.983)
			21	80	11.4		
Lu RL (2014)	Retrospective	Gefitinib or erlotinib	19	42	14.2	<0.05^d^	0.676 (0.4570–1.0000)
			21	34	9.6		
Choi CM (2014)	Retrospective	Gefitinib 250 mg/day, po or erlotinib 150 mg/day, po	19	77	NA	NA	0.846 (0.2815–2.5437)
			21	43	NA		

a Exon of EGFR mutation means either exon 19 deletion or exon 21 L858R mutation.

b HR_19/21_ of TKI represents HR_19 exon deletion/exon 21 L858R mutation_ in TKI therapy cohort.

c We considered time to progression (TTP) as PFS in studies of Asahina H and Jackman DM.

d We considered *P*-value as 0.05 in Lu RL's study to calculate the HR_19/21_ of TKI for PFS and its 95% CI.

Abbreviations: CI  =  confidence interval; EGFR  =  epidermal growth factor receptor; HR  =  Hazard ratio; NA  =  not available; PFS  =  progression-free survival; TKI  =  tyrosine kinase inhibitor.

### Indirect meta-analysis of EGFR exon 19 deletions vs. exon 21 L858R mutations under TKI therapy for PFS

**Figure 2:**
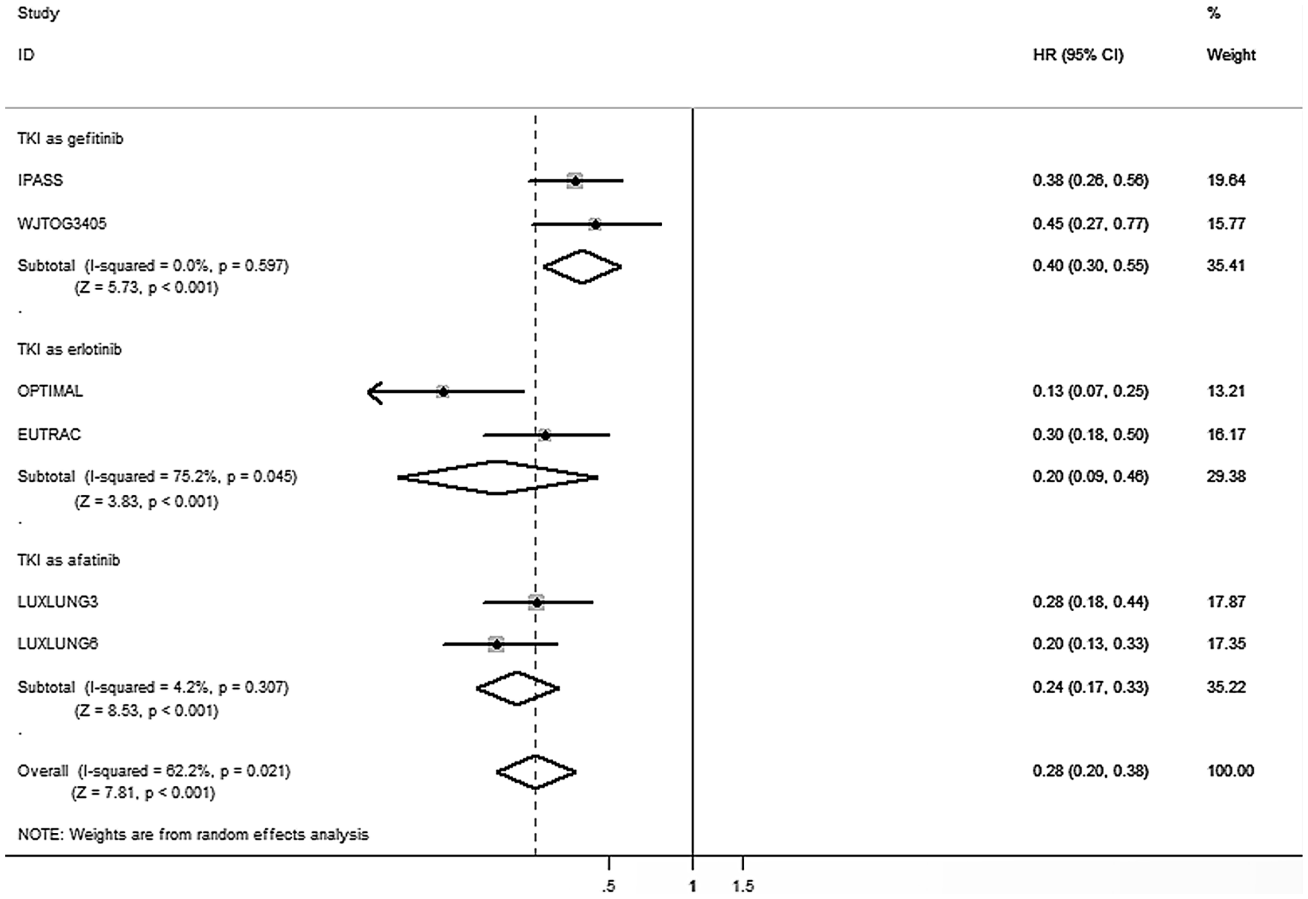
Direct comparison of TKI versus chemotherapy in EGFR exon 19 deletions cohort in terms of HR for PFS. CI  =  confidence interval; EGFR  =  epidermal growth factor receptor; HR  =  Hazard ratio; PFS  =  progression-free survival; TKI  =  tyrosine kinase inhibitor.

**Figure 3:**
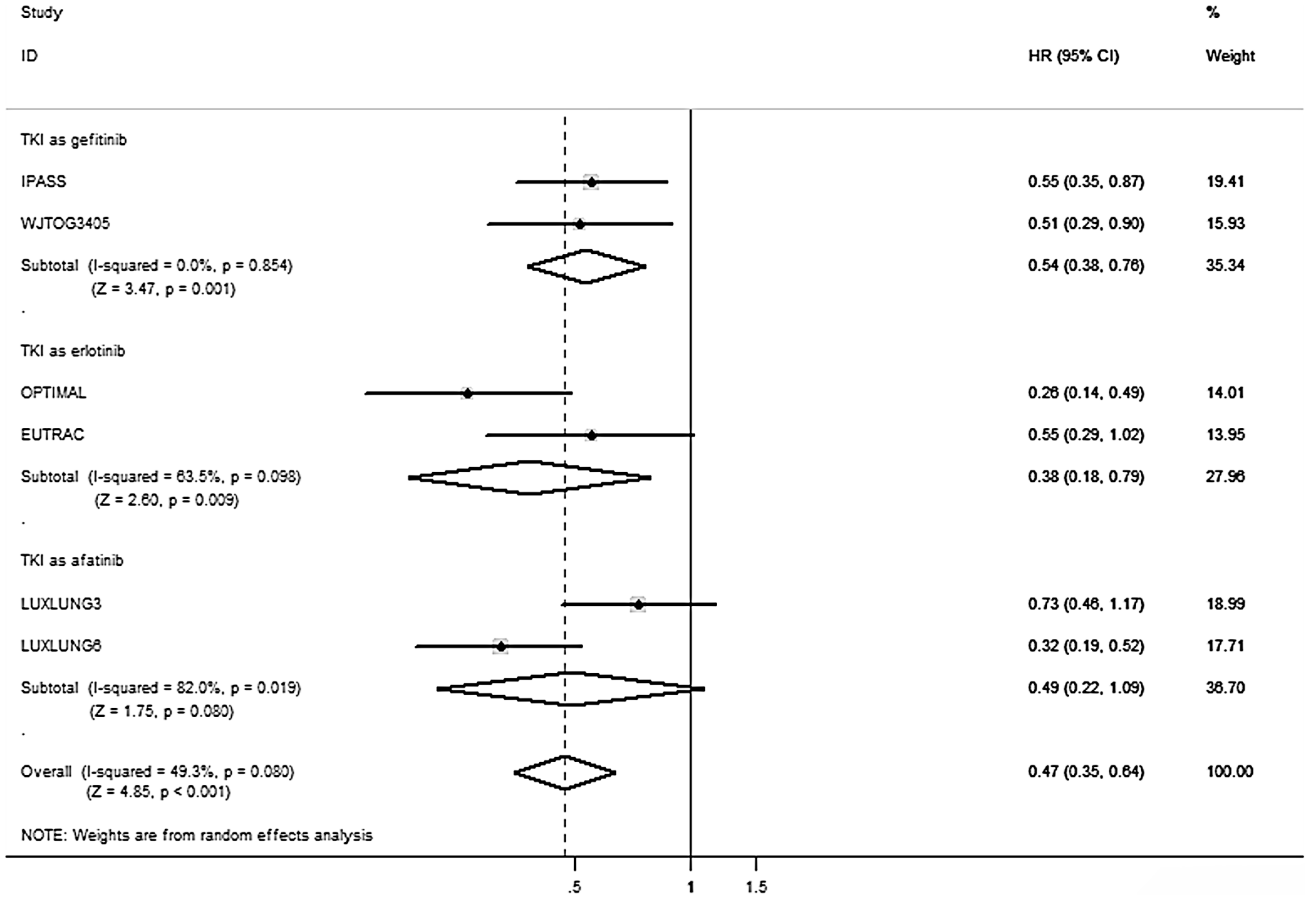
Direct comparison of TKI versus chemotherapy in EGFR exon 21 L858R mutations cohort in terms of HR for PFS. CI  =  confidence interval; EGFR  =  epidermal growth factor receptor; HR  =  Hazard ratio; PFS  =  progression-free survival; TKI  =  tyrosine kinase inhibitor.

**Figure 4:**
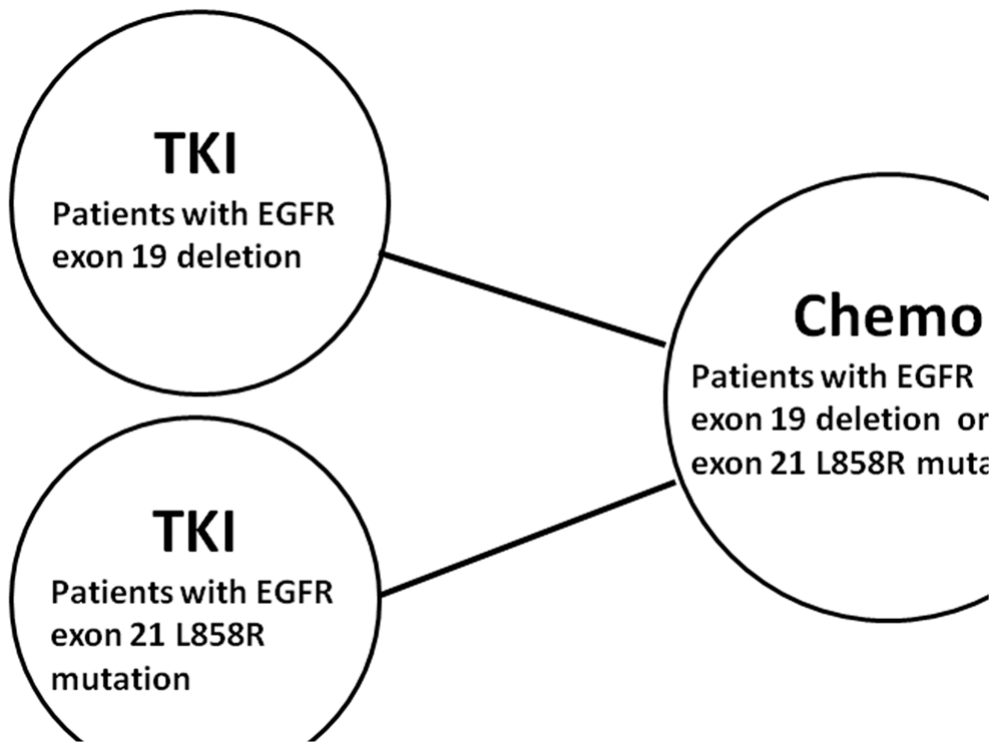
Geometric distribution of indirect comparisons. Solid lines between regimens represented the existence of direct comparisons. Chemo  =  chemotherapy; EGFR  =  epidermal growth factor receptor; TKI  =  tyrosine kinase inhibitor.

For advanced NSCLC patients with EGFR exon 19 deletion, the pooled HR of EGFR-TKIs compared to conventional chemotherapy were 0.28 (95% CI, 0.20–0.38, *P*<0.001). For another sensitive EGFR mutation type, L858R mutation in exon 21, the pooled HR were 0.47 (95% CI 0.35 to 0.64, *P*<0.001). In addition, subgroup analyses revealed similar results that both exon 19 deletion (HR_gefitinib/chemotherapy_ = 0.40, 95% CI 0.30 to 0.55, *P*<0.001; HR_erlotinib/chemotherapy_ = 0.20, 95% CI 0.09 to 0.46, *P*<0.001; HR_afatinib/chemotherapy_ = 0.24, 95% CI 0.17 to 0.33, *P*<0.001) and exon 21 L858R mutation (HR_gefitinib/chemotherapy_ = 0.54, 95% CI 0.38 to 0.76, *P* = 0.001; HR_erlotinib/chemotherapy_ = 0.38, 95% CI 0.18 to 0.79, *P* = 0.009; HR_afatinib/chemotherapy_ = 0.49, 95% CI 0.22 to 1.09, *P* = 0.080) resulted in reduced progression risk through different types of TKIs (**Figure 2** and **Figure 3**). **Figure 4** shows the relationship of the indirect comparisons. Present evidence suggested that there was little difference in the efficacy of platinum-based doublet for patients with exon 19 deletion and those with exon 21 L858R mutation. Indirect comparison revealed that patients with exon 19 deletion had more favorable outcome for PFS than those with exon 21 L858R mutation (HR_19/21_ = 0.59, 95% CI 0.38 to 0.92; *P* = 0.019) under TKIs therapy. Besides, we found similar results through subgroup analyses stratified by TKI types (Gefitinib: HR_19/21_ = 0.76, 95% CI 0.47 to 1.21, *P* = 0.244; Erlotinib: HR_19/21_ = 0.53, 95% CI 0.18 to 1.61, *P* = 0.264; Afatinib: HR_19/21_ = 0.49, 95% CI 0.21 to 1.17, *P* = 0.108) (**Table 3**).

**Table 3:** Indirect comparison of EGFR exon 19 deletion versus EGFR exon 21 L858R mutation in TKI therapy cohort in terms of HR for PFS.

TKI	HR_19/21_ of TKI^a^ for PFS (95% CI)	P-value
Gefitinib	0.76 (0.47–1.21)	0.244
Erlotinib	0.53 (0.18–1.61)	0.264
Afatinib	0.49 (0.21–1.17)	0.108
Overall	0.59 (0.38–0.92)	0.019

a HR_19/21_ of TKI represents HR_19 exon deletion/exon 21 L858R mutation_ in TKI therapy cohort.

Abbreviations: CI  =  confidence interval; EGFR  =  epidermal growth factor receptor; HR  =  Hazard ratio; PFS  =  progression-free survival; TKI  =  tyrosine kinase inhibitor.

### Direct meta-analysis of EGFR exon 19 deletions vs. exon 21 L858R mutations under TKI therapy for PFS

**Figure 5:**
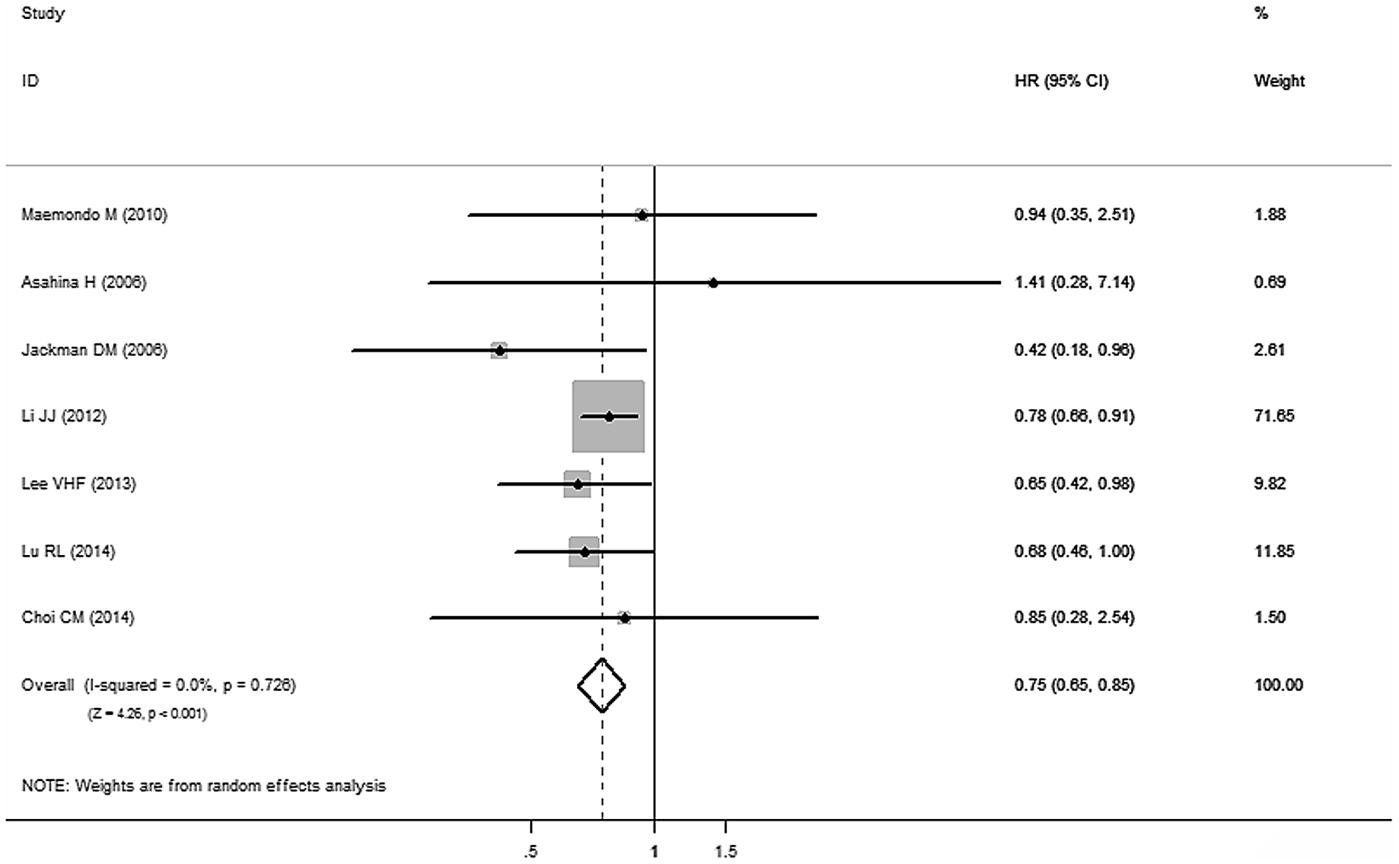
Direct comparison of EGFR exon 19 deletions versus EGFR exon 21 L858R mutations in TKI therapy cohort in terms of HR for PFS. CI  =  confidence interval; EGFR  =  epidermal growth factor receptor; HR  =  Hazard ratio; PFS  =  progression-free survival; TKI  =  tyrosine kinase inhibitor.

Based on the data from the above seven studies [8], [11], [13], [26], [27], [28], [29], advanced NSCLC patients with exon 19 deletion benefited from longer PFS than those with exon 21 L858R mutation (HR_19/21_ = 0.75, 95% CI 0.65 to 0.85; *P*<0.001) (**Figure 5**). There was no publication bias for outcome measures, as the funnel plot analysis showed a symmetrical appearance and all *p* values were greater than 0.05 in Begg's test and Egger's test.

## Discussion

For patients with advanced NSCLC, the association of specific EGFR mutation genotype and the efficacy or prognosis of first-line EGFR-TKIs therapy remains to be unclear. A meta-analysis incorporating all available data from correlative studies was a good way to address this question. We conducted this study and found that patients with exon 19 deletion had significantly reduced disease progression risk than those with exon 21 L858R mutation after front-line TKIs. Additionally, similar trends of favorable outcome of PFS in exon 19 deletion among different first-line EGFR-targeted agents (gefitinib, erlotinib and afatinib), were presented in our work, but the statistical significances were not approached.

Our results derived three interpretations. Firstly, exon 19 deletions might be more efficiently inhibited by EGFR-TKIs than exon 21 L858R mutations[8].Mutant forms of exon 19 deletion and L858R mutation had a reduced affinity for its natural substrate, ATP, but equivalent or increased affinity for reversible EGFR-TKIs, which explained why tumors with these EGFR mutations had higher sensitivity to TKIs [30], [31]. If exon 19 deletion results in EGFR structural alterations which bind TKIs more tightly than L858R mutation, it could support our hypothesis. However, an in vitro study showed that the growth of NSCLC cell lines harboring exon 19 deletion or L858R mutation were almost equally inhibited by equivalent concentrations of gefitinib, and the degree of EGFR phosphorylation [32].

Secondly, T790M mutation, which was associated with acquired resistance to reversible EGFR-TKIs [33], might occur more frequently in L858R mutation. However, a prospective rebiopsy protocol among different EGFR mutated patients with acquired resistance to EGFR-TKIs showed that no difference between exon 19 deletions (63%) and L858R mutations (61%) existed in terms of the presence of T790M mutation [34]. Another recent phase II trials found similar results [35].

Thirdly, exon 21 L858R mutation co-existing with other uncommon mutations might affect the hypersensitivity of L858R to EGFR-TKIs. A retrospective study which investigated the frequency of complex mutations in 783 NSCLC patients found the majority was G719S plus L858R mutation (n = 8), the result also suggested poor response to gefitinib in patients with G719S plus L858R mutation [36]. Besides, previous studies revealed that patients with exon 18 (G719S) plus L858R mutation showed poor outcomes with gefitinib [37], [38], [39].

Based on the above hypotheses, the mechanism of more favorable efficacy in exon 19 deletion compared with L858R mutation is still controversial. Nonetheless, regardless of what the true causes might be, this comprehensive analysis statistically confirmed that patients with exon 19 deletion were associated with longer PFS compared to those with L858 mutation at exon 21. The result leads to some important hints. Firstly, we suggest that investigators should consider the proportion of type of sensitive EGFR mutation as a stratification factor in designing or reviewing clinical studies involving target therapy. In addition, the mechanism of EGFR-TKIs acting on different genotypes of EGFR mutants is more complicated than we imagined before. Therefore, more efforts should be made to investigate the pharmacological essence inside.

One research reported that patients with EGFR-mutant after front-line TKIs might develop as acquired resistance to TKI [20]. Besides, some previous investigations found inferior response to EGFR-TKIs following treatment of chemotherapy [40], [41]. According to previous evidence, getting second-line or third-line EGFR-TKIs involved will tangle the discussion. Therefore, in order to minimize the crossover effects, we only focused on first-line EGFR-TKIs in our analyses.

This is the first study to comprehensively answer the impact of different EGFR mutation types on TKIs. However, there existed several limitations. First, our meta-analysis was based on subgroup data of included articles, which might compromise the evidence level. In addition, the small number of included studies negated statistically significances in subgroup analyses. Finally, our work was conducted based on the assumption that there were no significant differences in the efficacy of platinum-based chemotherapy between exon 19 deletion and L858R mutation for advanced NSCLC patients. Few studies focused on the prognostic value of different EGFR mutation in patients with advanced NSCLC with chemotherapy [42], and as a consequence, our hypothesis is in need of confirmation by more convincing evidence.

In conclusion, for patients with advanced NSCLC, EGFR exon 19 deletion might be associated with longer PFS than those harboring L858 mutation at exon 21 after first-line EGFR-TKIs. Sensitive EGFR mutation type should be considered an essential factor in studies regarding EGFR-targeted agents.

## Clinical Practice Points

The association of specific EGFR mutation genotype with the prognosis in patients with advanced NSCLC after first-line EGFR-TKIs was controversial based on previous reports. This comprehensive analysis statistically confirmed exon 19 deletions were associated with longer PFS compared with L858 mutations at exon 21. Additionally, the superior benefits of PFS in former genotype of EGFR mutation showed similar trends among different EGFR-targeted agents (gefitinib, erlotinib and afatinib), although the significances were not approached statistically. The result gave us some important hints. Firstly, we strongly suggested that investigators should consider the proportion of type of sensitive EGFR mutation as a stratification factor in designing or reviewing clinical studies regarding target therapy. In addition, the mechanism of EGFR-TKIs acting on different genotypes of EGFR mutants was more complicated than what we acknowledged. Therefore, more efforts should be made to investigate pharmacological essence inside.

## Supporting Information

Checklist S1 **PRISMA Checklist.**
(DOC)
