# Supplementary material for: Patients with Exon 19 Deletion Were Associated with Longer Progression-Free Survival Compared to Those with L858R Mutation after First-Line EGFR-TKIs for Advanced Non-Small Cell Lung Cancer: A Meta-Analysis
Source: PLoS One. 2014 Sep 15;9(9):e107161. doi: 10.1371/journal.pone.0107161 (PMC4164616; doi:10.1371/journal.pone.0107161)
Supplement: Checklist S1 — PRISMA Checklist. (DOC) [file pone.0107161.s001.doc]

| **Section/topic** | **#** | **Checklist item** | **Reported on page #** |
| --- | --- | --- | --- |
| **TITLE** | | |  |
| Title | 1 | Exon 19 deletions were associated with longer PFS compared to L858R mutations at exon 21 in treatment with first-line EGFR-TKIs: A meta-analysis | Title |
| **ABSTRACT** | | |  |
| Structured summary | 2 | Backgrounds: The superior efficacy of epidermal growth factor receptor-tyrosine kinase inhibitors (EGFR-TKIs) compared with cytotoxic chemotherapy in patients harboring sensitive EGFR mutations has been extensively proved by a series of trials. However, the question of whether the efficacy of EGFR-TKIs differs between EGFR exon 19 deletions and exon 21 L858R mutations has not been statistically answered.  Methods: Subgroup data on hazard ratio (HR) for progression free survival (PFS) in correlative studies were extracted and were synthesized based on random-effect model. Comparison of outcomes between specific mutations was estimated through indirect and direct methods, respectively.  Results: A total of 13 studies which reported advanced NSCLC patients with either 19 or 21 exon alteration receiving first-line EGFR-TKIs were included. Based on data of six clinical trials for indirect meta-analysis, the pooled HRTKI/chemotherapy for PFS were 0.28 (95% CI 0.20-0.38, P < 0.001) in patients with 19 exon deletions and 0.47 (95% CI 0.35-0.64, P < 0.001) in those with exon 21 L858R mutations. Indirect comparison revealed that patients with exon 19 deletions had more favorable outcome for PFS than those with exon 21 L858R mutations (HR19 exon deletions/exon 21 L858R mutations = 0.59, 95% CI 0.38-0.92; P = 0.019). Additionally, direct meta-analysis showed similar result (HR19 exon deletions/exon 21 L858R mutations = 0.75, 95% CI 0.65 to 0.85; P < 0.001) by incorporating direct comparison results from another seven studies.  Conclusions: Exon 19 deletions might be associated with longer PFS compared with L858 mutations at exon 21.  Keywords: NSCLC, EGFR, mutation, First-line TKI, meta-analysis | Abstract |
| **INTRODUCTION** | | |  |
| Rationale | 3 | The superior efficacy of epidermal growth factor receptor-tyrosine kinase inhibitors (EGFR-TKIs) compared with cytotoxic chemotherapy in patients harboring sensitive EGFR mutations has been extensively proved by a series of trials. However, the question of whether the efficacy of EGFR-TKIs differs between EGFR exon 19 deletions and exon 21 L858R mutations has not been statistically answered. | Introduction |
| Objectives | 4 | To evaluate whether the clinical outcome differs between EGFR exon 19 deletions and exon 21 L858R mutations in advanced NSCLC patients treated with front-line EGFR-TKIs. | Introduction |
| **METHODS** | | |  |
| Protocol and registration | 5 | The data collection and assessment of methodological quality followed the QUORUM and the Cochrane Collaboration guidelines (http://www.cochrane.de). | Methods |
| Eligibility criteria | 6 | Eligible studies should meet the following criteria: (i) clinical trials or retrospective studies which investigated or reported a subset of NSCLC patients with specific sensitive EGFR mutation (exon 19 deletions or exon 21 L858R mutations) who received first-line monotherapy of EGFR-TKIs (e.g. gefitinib, erlotinib or afatinib) mainly in local advanced or metastatic (IIIB or IV) stage; (ii) EGFR mutation analysis was performed on available tumor tissue samples instead of circulating free DNA in serum; (iii) prior neoadjuvant or adjuvant chemotherapy in patients with recurrence after surgery was permitted if it had elapsed from last administration to relapse at least 6-month; (iv) hazard ratios (HRs) of EGFR-TKIs compared with conventional chemotherapy for progression-free survival (PFS) using subgroup analysis stratified for types of sensitive EGFR mutation or HRs of exon 19 deletions compared with exon 21 L858R mutations for PFS in terms of EGFR-TKIs were available. | Methods |
| Information sources | 7 | All relevant articles were retrieved by searching PubMed, Embase and the Central Registry of Controlled Trials of the Cochrane Library. | Methods |
| Search | 8 | All relevant articles were retrieved by using a combination of the terms “EGFR”, “epidermal growth factor receptor”, “tyrosine kinase inhibitors”, “TKI”, “exon”, “mutation”, “non-small-cell lung cancer” and “NSCLC”. An additional search through Google Scholar and a manual search through reference lists of relevant reviews and included studies were additionally performed. No restriction by language or year was set in the search. | Methods |
| Study selection | 9 | We identified 521 records according to the search strategy and focused on 13 eligible studies which reported advanced NSCLC patients with either 19 or 21 exon alteration who received first-line monotherapy of EGFR-TKIs. | Methods |
| Data collection process | 10 | Two authors (ZY and SJ) carried out the search independently. The clinical outcome for this meta-analysis was PFS. The data collection and assessment of methodological quality followed the QUORUM and the Cochrane Collaboration guidelines (http://www.cochrane.de). | Methods |
| Data items | 11 | Data of PFS were extracted as the HR and its 95% confidence interval (CI) from subgroup analysis by two investigators (KS and FW) independently. We used median PFS and the P-value to calculate the HR and its 95% CI them through REVIEW MANAGER (version 5.1 for Windows; the Cochrane Collaboration, Oxford, UK) if they were not displayed directly. | Methods |
| Risk of bias in individual studies | 12 | An extensive search strategy was made to minimize the potential for publication bias. Graphical funnel plots were generated to visually assess a publication bias. The statistical methods to detect funnel plot asymmetry were the rank correlation test of Begg and Mazumdar and the regression asymmetry test of Egger. | Methods |
| Summary measures | 13 | Data of PFS were extracted as principal summary measures. Pooled HRs for PFS with 95% CI were calculated. | Methods |
| Synthesis of results | 14 | Pooled HRs for PFS with 95% CI were calculated. Heterogeneity across studies was assessed with a forest plot and the inconsistency statistic (I2). Random-effects model was employed in case of potential heterogeneity and to avoid underestimation of standard errors of pooled estimates in direct meta-analyses as well as subsequent indirect comparison. All calculations were performed using STATA 11.0 (StataA Corp, College Station, TX). | Methods |

Page 1 of 2

| **Section/topic** | **#** | **Checklist item** | **Reported on page #** |
| --- | --- | --- | --- |
| Risk of bias across studies | 15 | Graphical funnel plots were generated to visually assess a publication bias. | Methods |
| Additional analyses | 16 | No additional analyses were performed. | Methods |
| **RESULTS** | | |  |
| Study selection | 17 | 521 studies were screened and assessed for eligibility, and finally included 17 articles in the review, with reasons for exclusions at each stage presented in a flow diagram as Figure 1. | Results |
| Study characteristics | 18 | Six phase III RCTs (IPASS20, WJTOG340521, OPTIMAL22, EUTRAC23, LUXLUNG324, and LUXLUNG625) which investigated the therapeutic effect of EGFR-TKIs (gefitinib, erlotinib and afatinib) compared with conventional chemotherapy (platinum-based doublet) for PFS using subgroup analysis stratified for types of sensitive EGFR mutation in 1382 advanced NSCLC chemo-naïve patients were selected for indirect meta-analysis. Another seven studies8,11,13,26-29 (clinical trials or retrospective studies) involving 549 advanced NSCLC EGFR mutants receiving first-line EGFR-TKIs (gefitinib or erlotinib) with direct comparison of exon 19 deletions and exon 21 L858R mutations for PFS were used for direct meta-analysis. | Results |
| Risk of bias within studies | 19 | There was no publication bias for outcome measures, with asymmetrical appearance on funnel plot analysis and all p values greater than 0.05 in Begg’s test and Egger’s test. | Results |
| Results of individual studies | 20 | Table 1 and Table 2 summarized the characteristics of involved studies for indirect meta-analysis and direct meta-analysis, respectively. | Results |
| Synthesis of results | 21 | According to available data on above six trials, we found the pooled HR of EGFR-TKIs compared with conventional chemotherapy for PFS were 0.28 (95% CI, 0.20-0.38, P < 0.001) in advanced NSCLC patients with EGFR 19 exon deletions and 0.47 (95% CI 0.35 to 0.64, P < 0.001) in those with EGFR exon 21 L858R mutations. In addition, subgroup analyses revealed similar results that patients with either 19 exon deletions (HRgefitinib/chemotherapy = 0.40, 95% CI 0.30 to 0.55, P < 0.001; HRerlotinib/chemotherapy = 0.20, 95% CI 0.09 to 0.46, P < 0.001; HRafatinib/chemotherapy = 0.24, 95% CI 0.17 to 0.33, P < 0.001) or exon 21 L858R mutations (HRgefitinib/chemotherapy = 0.54, 95% CI 0.38 to 0.76, P = 0.001; HRerlotinib/chemotherapy = 0.38, 95% CI 0.18 to 0.79, P = 0.009; HRafatinib/chemotherapy = 0.49, 95% CI 0.22 to 1.09, P = 0.080) obtained superior benefit from different types of TKIs compared with platinum-based chemotherapy in terms of PFS (Figure 2 and Figure 3).  Indirect comparison revealed that patients with exon 19 deletions had more favorable outcome for PFS than those with exon 21 L858R mutations (HR19/21 = 0.59, 95% CI 0.38 to 0.92; P = 0.019) under TKIs therapy based on overall results. Besides, we found approximate results through subgroup analyses stratified by TKI types (Gefitinib: HR19/21 = 0.76, 95% CI 0.47 to 1.21, P = 0.244; Erlotinib: HR19/21 = 0.53, 95% CI 0.18 to 1.61, P = 0.264; Afatinib: HR19/21 = 0.49, 95% CI 0.21 to 1.17, P = 0.108) with insignificant P-value statistically (Table 3). advanced NSCLC patients with exon 19 deletions had better outcome for PFS than those with exon 21 L858R mutations (HR19/21 = 0.75, 95% CI 0.65 to 0.85; P < 0.001) under EGFR-TKIs therapy (Figure 5). | Results |
| Risk of bias across studies | 22 | There was no publication bias for outcome measures, with asymmetrical appearance on funnel plot analysis and all p values greater than 0.05 in Begg’s test and Egger’s test. | Results |
| Additional analysis | 23 | No. |  |
| **DISCUSSION** | | |  |
| Summary of evidence | 24 | Based on data of six clinical trials for indirect meta-analysis, the pooled HRTKI/chemotherapy for PFS were 0.28 (95% CI 0.20-0.38, P < 0.001) in patients with 19 exon deletions and 0.47 (95% CI 0.35-0.64, P < 0.001) in those with exon 21 L858R mutations. Indirect comparison revealed that patients with exon 19 deletions had more favorable outcome for PFS than those with exon 21 L858R mutations (HR19 exon deletions/exon 21 L858R mutations = 0.59, 95% CI 0.38-0.92; P = 0.019). Additionally, direct meta-analysis showed similar result (HR19 exon deletions/exon 21 L858R mutations = 0.75, 95% CI 0.65 to 0.85; P < 0.001) by incorporating direct comparison results from another seven studies. | Results |
| Limitations | 25 | First, our meta-analysis was based on subgroup data extracted from included studies, which somehow compromised the evidence level. In addition, the small number of included studies negated statistically significances in subgroup analyses. Finally, our work was conducted based on the assumption that no significant efficacy difference existed between patients with exon 19 deletions and exon 21 L858R mutations in platinum-based doublet. As a consequence of few studies focus on the the prognostic value of different EGFR Mutation in Patients with NSCLC undergoing platinum-based chemotherapy 43, our hypothesis remains to be confirmed by more convicing evidence. | Discussion |
| Conclusions | 26 | This meta-analysis showed that advanced NSCLC patients with exon 19 deletions might be associated with longer PFS compared with those harboring L858 mutations at exon 21.  Sensitive EGFR mutation type should be considered an essential factor in studies regarding EGFR-targeted agents. | Discussion |
| **FUNDING** | | |  |
| Funding | 27 | The work had four fundings. | Funding |

*From:*  Moher D, Liberati A, Tetzlaff J, Altman DG, The PRISMA Group (2009). Preferred Reporting Items for Systematic Reviews and Meta-Analyses: The PRISMA Statement. PLoS Med 6(6): e1000097. doi:10.1371/journal.pmed1000097

For more information, visit: **www.prisma-statement.org**.

Page 2 of 2
